# Phylogenetics of *Leptocereus* (Cactaceae) on Hispaniola: clarifying species limits in the *L. weingartianus* complex and a new species from the Sierra de Bahoruco

**DOI:** 10.3897/phytokeys.172.59497

**Published:** 2021-01-29

**Authors:** Lucas C. Majure, Yuley Encarnación, Teodoro Clase, Brígido Peguero, Kelly Ho, Duniel Barrios

**Affiliations:** 1 University of Florida Herbarium (FLAS), Florida Museum of Natural History, Gainesville, FL 32611, USA; 2 Department of Biology, University of Florida, Gainesville, Florida, USA; 3 Departamento de Botánica, Jardín Botánico Nacional “Dr. Rafael M. Moscoso”, Santo Domingo, Dominican Republic; 4 Grupo de Ecología y Conservación, Jardín Botánico Nacional, Universidad de La Habana, Habana, Cuba

**Keywords:** Biodiversity, Greater Antilles, plastome phylogeny, Seasonally Dry Tropical Forest

## Abstract

The Antillean genus *Leptocereus* represents an *in-situ* radiation among the Greater and Lesser Antilles of 19 currently recognized species. Extensive fieldwork carried out in the Dominican Republic over recent years has revealed that the species limits of *Leptocereus* of Hispaniola are more complex than previously thought. There are four currently recognized species that occur on the island, *L. demissus*, *L. paniculatus*, *L. undulosus* and *L. weingartianus*. We evaluate species limits in this group based on DNA sequence data and phylogenetic analysis, morphological characters and a survey of herbarium specimens from across Hispaniola. Based on our analyses, it is clear that at least five species occur on the island of Hispaniola, with the new species from Sierra de Bahoruco, *L. velozianus*, described here. We provide an identification key, distribution maps and photographic plates for all species on Hispaniola based on our own fieldwork and the study of herbarium specimens. The description of yet another species of *Leptocereus* on Hispaniola reiterates the importance of the poorly studied, but yet biodiverse, seasonally dry tropical forest in the Antilles.

## Introduction

Seasonally dry tropical forests (SDTF) are one of the most threatened forest types on the planet with only around 10% of their original coverage still remaining intact in the Neotropics (Banda-R. et al. 2016) mostly owing to anthropogenic pressures, such as charcoal production and agriculture (Pennington et al. 2000, 2004). This forest type is extensive throughout the Greater Antilles; however, it has been poorly studied (Gentry 1982; Pennington et al. 2005) compared to other forest types on the islands or dry forest from other parts of the Neotropics. Recent work has revealed that these understudied forests have much undocumented biodiversity (e.g., Mejía and García 1997; Fryxell and Clase 2007; Areces-Mallea 2017, 2018; Majure et al. 2020; Martínez-Gordillo et al. 2020). Cactaceae are a conspicuous element of the diverse SDTF of the Greater Antilles, with roughly 94 taxa occurring in the region (Majure et al. unpubl. data). An estimated 35 species occur on the island of Hispaniola, including the Caribbean endemic *Leptocereus* (A. Berger) Britton & Rose. *Leptocereus* s.l. is a clade of 19 currently recognized species of trees and erect to sprawling shrubs, and represents an *in-situ* Antillean radiation that occurs in SDTF, primarily of the Greater Antilles, where it is most diverse, but also occurs in the Lesser Antilles on the island of Anegada (Barrios et al. 2020). Recent phylogenetic work by Barrios et al. (2020) showed that the two large tree species endemic to Cuba and Hispaniola, and traditionally circumscribed in the genus *Dendrocereus* (Britton and Rose 1920; Anderson 2001; Hunt et al. 2006), formed a monophyletic group [the *Dendrocereus* clade (D)] clearly nested within *Leptocereus*. Barrios et al. (2020) also resolved two other primary clades in *Leptocereus*, the Cuban (CU) clade consisting of species endemic to Cuba, and the Hispaniolan-Puerto Rican (EPR) clade consisting of taxa endemic to Hispaniola, Puerto Rico and outlying islands.

On Hispaniola, three species of *Leptocereus* (including *Dendrocereus*) were formerly recognized (*L. paniculatus* (Lam.) D. R. Hunt, *L. undulosus* (DC.) D. Barrios & Majure, and *L. weingartianus* E. Hartmann), and Areces-Mallea (2017) recently described a fourth species (*L. demissus* Areces) from southwestern Dominican Republic from the SDTF south of the Sierra de Bahoruco mountain range. Thus, currently, four species are recognized on Hispaniola.

Barrios et al. (2020) showed that the Hispaniolan endemic, *Leptocereus weingartianus*, was sister to a clade containing another Hispaniolan endemic, *L. paniculatus*, and a clade of two Puerto Rican species, *L. grantianus* Britton and *L. quadricostatus* Britton & Rose. *Leptocereus weingartianus* is widely distributed across Hispaniola and is morphologically heterogeneous, forming relatively large shrubs or small trees (Areces-Mallea 2017) with apical branches that are often sprawling among surrounding, dense vegetation of SDTF (Majure et al. pers. obsv.). The new species, *L. demissus*, described by Areces-Mallea (2017), is morphologically very similar to *L. weingartianus*, especially considering the sprawling growth form of ultimate stem segments (Majure et al. pers. obsv.), however, several reproductive characters appear to separate the two species morphologically (Areces-Mallea 2017). Recent fieldwork in the Sierra de Bahoruco has revealed populations of an unidentified taxon of what morphologically appeared to be part of the *L. weingartianus* complex, however, which differed from typical *L. weingartianus* in stem and spine features. Therefore, it was clear given the morphological heterogeneity of *L. weingartianus*, and the wide distribution and phenetic similarity of putative close relatives, such as *L. demissus*, that phylogenetic analyses of these taxa on Hispaniola were greatly needed.

We wanted to determine whether the morphologically disparate populations from the Sierra de Bahoruco were indeed closely related to the *L. weingartianus* complex, and whether or not the morphologically similar *L. demissus* was distinct from *L. weingartianus* based on phylogenetic relationships. We sampled multiple populations of typical *L. weingartianus*, one population of the newly described *L. demissus* and several populations of the new morphotype from Sierra de Bahoruco, as well as taxa from all major clades of *Leptocereus* (sensu Barrios et al. 2020) and carried out a phylogenetic reconstruction based on nearly entire plastome sequencing from genome skimming. We also reviewed herbarium specimens from across the distribution of *Leptocereus* on Hispaniola, and we herein provide distribution maps, photographic plates, and an identification key to all species on Hispaniola, as well as a description of the new species uncovered during this work.

## Materials and methods

All species of *Leptocereus* from Hispaniola, representing the EPR and D clades, as well as the new material from Sierra de Bahoruco, were sampled here for phylogeny reconstruction along with three taxa from the CU clade of *Leptocereus* and the Cuban *L. nudiflorus* (Britton & Rose) D.Barrios & S.Arias of the D clade. Likewise, *L. grantianus* and *L. quadricostatus* of the EPR clade were sampled. Outgroups included here were based on previous work by Barrios et al. (2020) and Majure et al. (unpubl. data) and included *Armatocereus*, *Calymmanthium*, *Cereus*, *Melocactus*, *Selenicereus*, and *Stenocereus* of Core Cactoideae (see Appendix 1).

Whole genomic DNAs of all taxa were extracted using a standard CTAB protocol with silica column cleaning (see Majure et al. 2019; Köhler et al. 2020). DNAs were resuspended in 300 ul of TE (Tris-EDTA) buffer (pH 8.0), and DNA quantity was analyzed on a Qubit 2.0 Fluorometer. Whole genomic DNAs were sent to Rapid Genomics LLC (http://rapid-genomics.com/home/; Gainesville, Florida, U.S.A.) for library preparation (including shearing) and sequencing via a genome skimming method, as in Majure et al. (2019). All taxa were sequenced on the Illumina HiSeq X platform using paired end reads (yielding 150 bp reads), and sixty samples were included per lane.

Raw reads of all taxa were imported in to Geneious (v. 11.1.5, Biomatters Ltd., Auckland, New Zeland) and reference-mapped using a previously, de-novo assembled partial plastome (including the large-single copy unit) of *Melocactus pedernalensis* M.M.Mejía & R.G.García (Majure, unpubl. data). Consensus sequences were then generated from reference-mapped plastomes, which were used for alignments. Sequence alignment was carried out using the MAFFT (Katoh and Standley 2016) plugin in Geneious. We analyzed our 104,697 bp dataset (including indels) under maximum likelihood (ML) with the RAxML (Stamatakis 2014) plugin in Geneious using the rapid bootstrapping algorithm and undertaking 100 bootstrap pseudoreplicates.

Specimens (ca. 50) from multiple herbaria (JBSD, NY, S, US), as well as those generated from our own fieldwork (DES, FLAS, JBSD), were consulted for determining the morphological distinctiveness of the new material from the Sierra de Bahoruco, as compared to the phenetically similar taxa *L. weingartianus* and *L. demissus*. Those data also were used to determine the distribution of all species of *Leptocereus* across Hispaniola and to generate our identification key to the species (in part).

## Results

**Figure 1.**
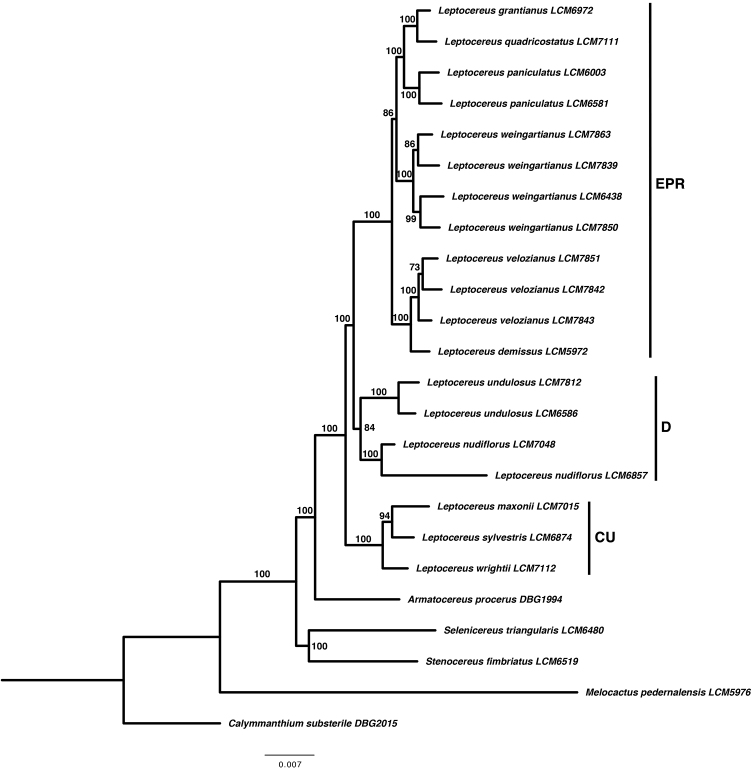
Phylogeny of *Leptocereus* with all major clades represented (sensu Barrios et al. 2020) and the new species *L. velozianus* included, resolved here as sister to *L. demissus*. Bootstrap values are given above the branches.

*Leptocereus* s.l. was resolved as monophyletic in our phylogenetic analysis, and all three principal subclades recovered by Barrios et al. (2020) were recovered here as well (i.e., CU, D, and EPR subclades)-all three subclades were mostly well-supported (CU, bs = 100%; D, bs = 84%, EPR, bs = 100%). The CU subclade was sister to a well-supported clade (bs = 100%) composed of the D and EPR subclades. Multiple accessions of the two species composing the D clade, *L. undulosus* and *L. nudiflorus* formed clades and were resolved as sister taxa in our topology, further demonstrating their phylogenetic distinctiveness. Within the EPR clade, a clade composed of *L. demissus* and the new species *L. velozianus* was sister to a clade composed of two subclades, the first formed by all accessions of *L. weingartianus* and the second by *L. paniculatus*, *L. grantianus* and *L. quadricostatus*. Thus, our results show that *L. weingartianus* is not closely related to the new species *L. velozianus*, but rather more closely related to the rest of the EPR clade. Likewise, although *L. demissus* appears to be phenetically more similar to *L. weingartianus*, it was more closely related to the new species *L. velozianus*. In all cases where multiple accessions were used per species, those species formed well-supported clades (Fig. 1).

**Figure 2.**
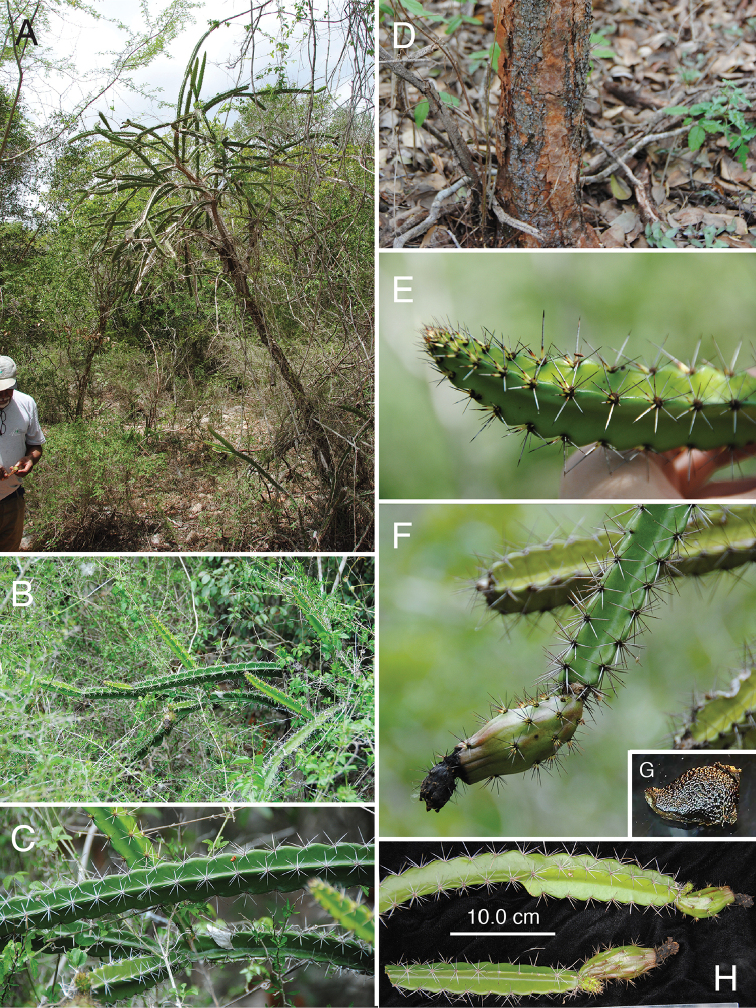
Photographic plate of *L. velozianus***A** small tree habit of *L. velozianus* alongside T. Clase for scale **B, C** arching branches and spine color **D** trunk and bark **E** growing stem showing white spines as they mature **F** spiny, immature fruit **G** colliculate-pitted seed, and **H** stems with immature fruit, showing crenate rib margins. **A, D, F, G** from *Majure 7851***B, C, H** from *Majure 7843*, and **E** from *Majure 7842*. Photos taken by L.C. Majure.

**Figure 3.**
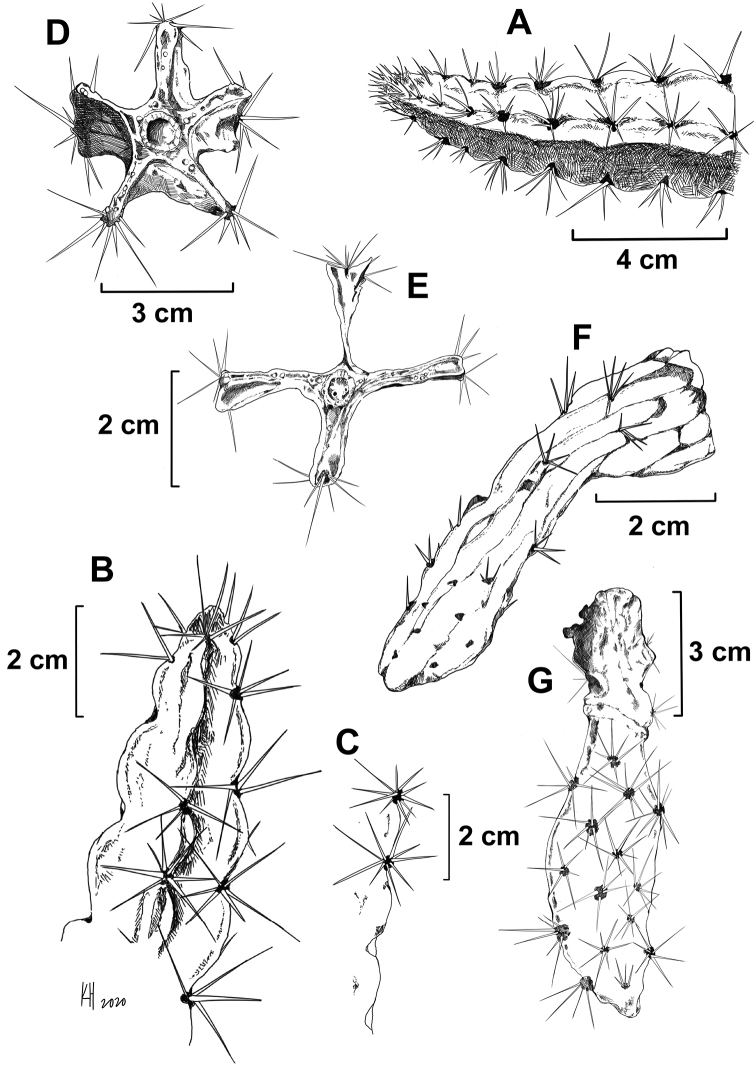
Illustration of *L. velozianus***A–C** stems showing crenate margins **D, E** cross sections showing 4-5 ribbed stems, also showing large crystals in the parenchyma **F** day old flower showing rounded external tepal apices and spiny pericarpel, and **G** immature fruit showing spiny pericarpel and remnant floral tube. **A** from *Majure 7842*, and **B–F** from *Majure 7843*.

**Figure 4.**
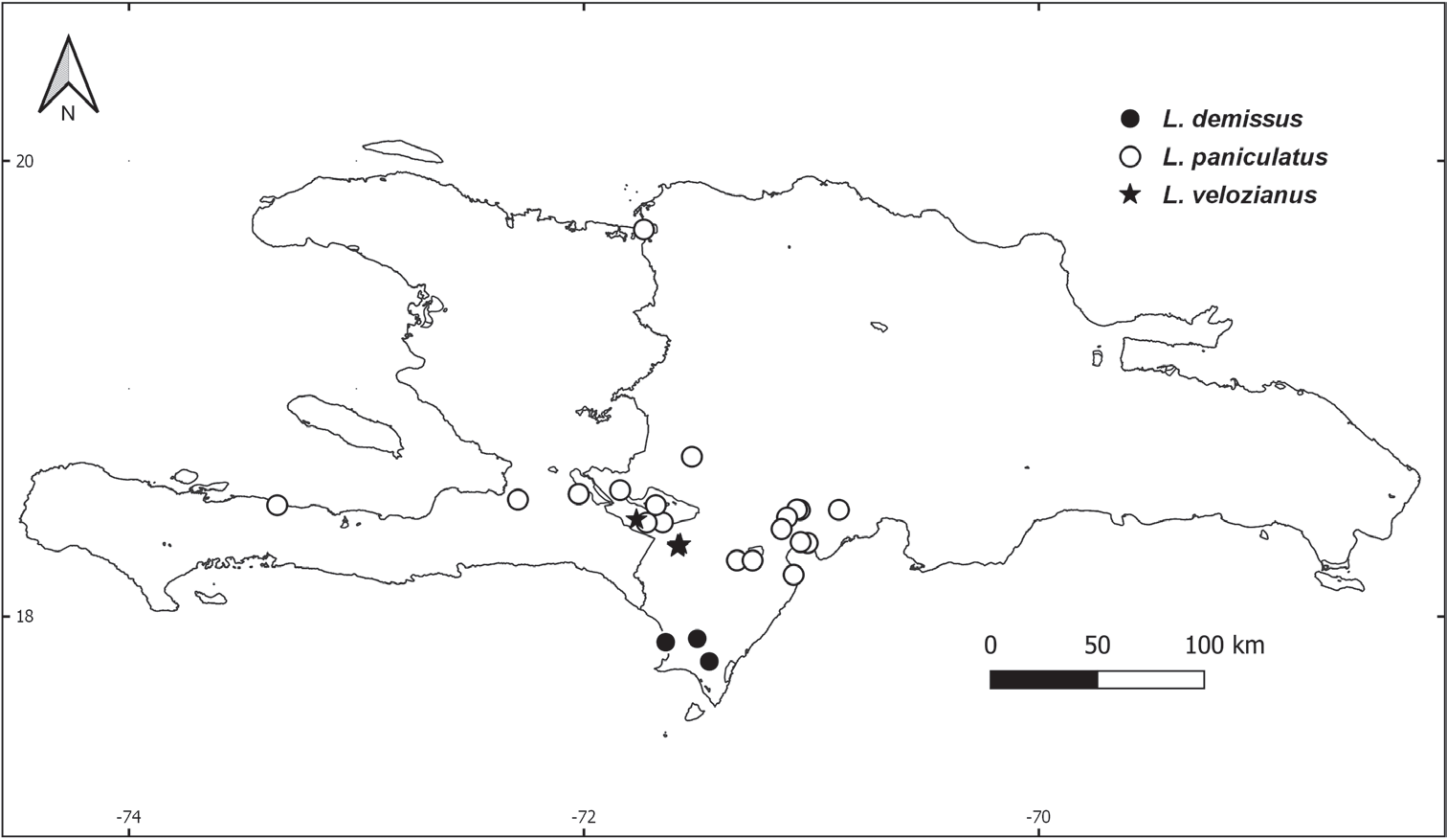
Distribution map of *L. demissus* (black circles), *L. paniculatus* (open circles), and *L. velozianus* (black stars) on Hispaniola.

**Figure 5.**
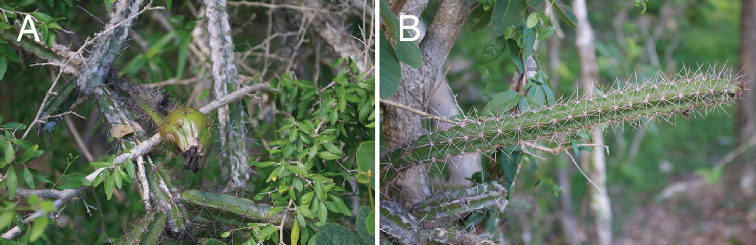
Photographic plate of *L. demissus***A** spiny fruit, and **B** slightly crenate stem of *L. demissus* (from *Majure 5972*). Photos by L.C. Majure.

**Figure 6.**
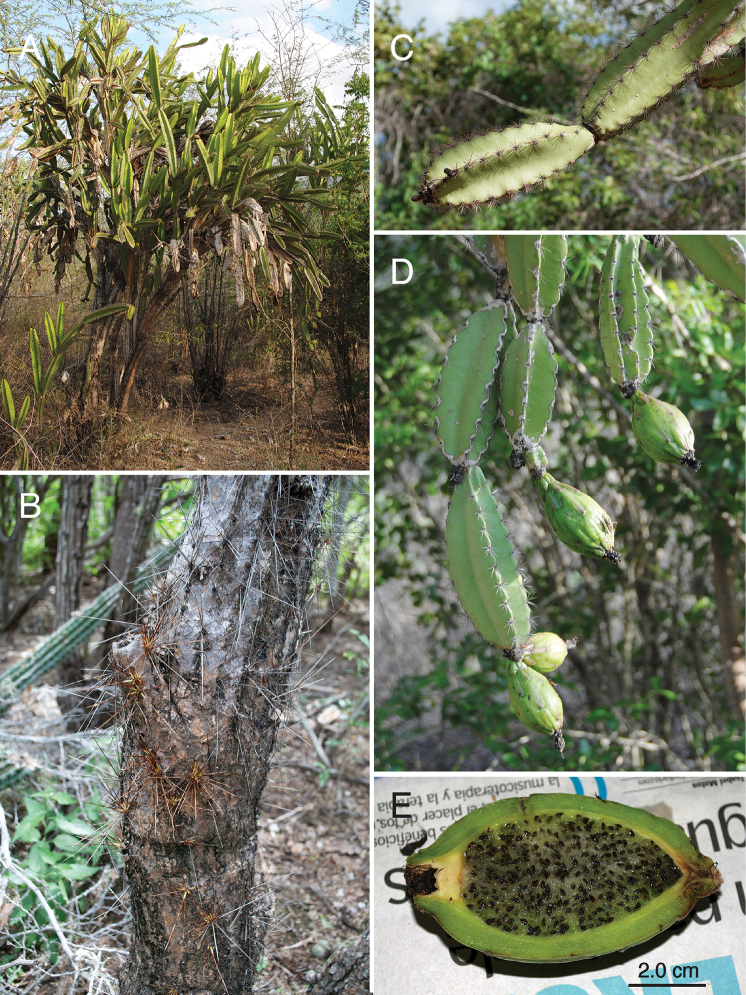
Photographic plate of *L. paniculatus***A** habit **B** spiny trunk **C** apical, spiny, four-angled stems **D** fruiting stems and tuberculate fruit, and **E** longitudinal section of mature fruit. **A, D** from *Majure 7817***B, C, E** from *Majure 6581*. Photos by L.C. Majure.

**Figure 7.**
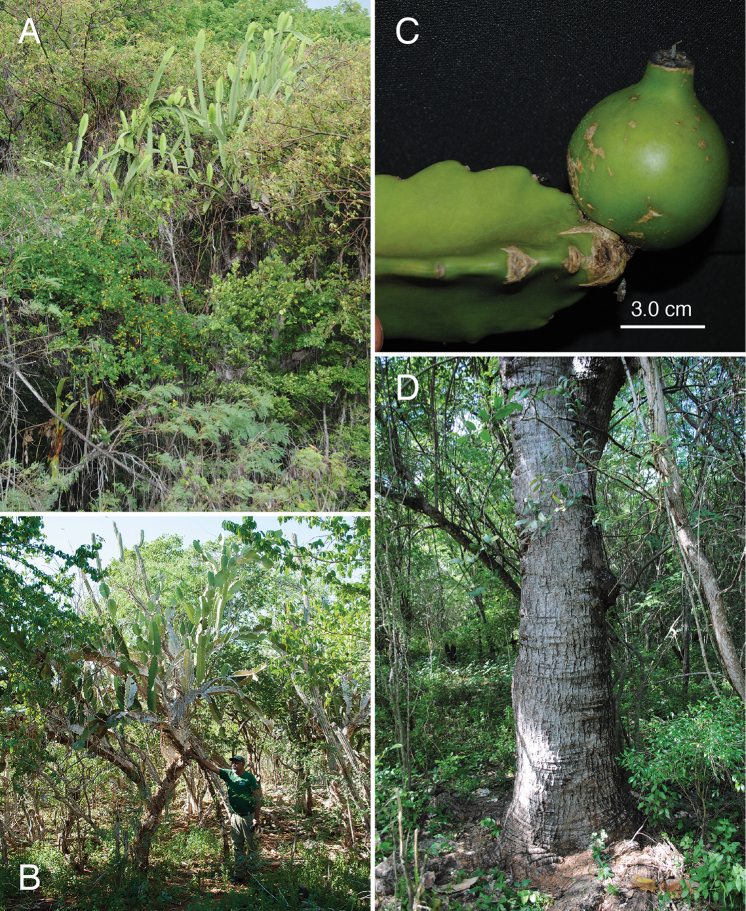
Photographic plate of *L. undulosus***A** large tree of *L. undulosus* erupting from surrounding tropical dry forest **B** small individual of *L. undulosus* with T. Clase for reference **C** fruit and three-angled stem **D** large, woody trunk (ca. >64 cm dbh). **A** from *Majure 7812***B, D** from *Majure. 6586* and **C** from *Majure 5974.* Photos by L.C. Majure.

*Leptocereus velozianus* (Figs 2, 3), which is restricted to the northwestern part of the Sierra de Bahoruco (Fig. 4), is slightly phenetically similar to the Puerto Rican species *L. quadricostatus*, given the crenate rib margins and spiny pericarpels, although those two species are not close relatives (Fig. 1). *Leptocereus velozianus* is most phenetically similar to two other Hispaniolan species (*L. weingartianus* and *L. demissus* – Figs 5, 9), especially *L. weingartianus*, but differs from them based on the spine color (white or cream versus yellow to yellowish-red, although, younger spines in *L. velozianus* can be yellow-cream colored with darker tips, thus slightly overlapping with *L. weingartianus*), and the overall size of the joints (1.2–3.5 cm in diameter in the latter two species and 2.7–3.7 in *L. velozianus*). *Leptocereus velozianus* has conspicuously crenate margins, generally more so than either *L. weingartianus* or *L. demissus*, although this is a variable character (Areces-Mallea 2017). Although Areces-Mallea (2017) mentioned that *L. demissus* has straight rib margins between areoles and *L. weingartianus* is more crenate, we found that character to be variable in both, with *L. weingartianus* also sometimes having nearly straight rib margins between areoles (Figs 5B, 9A). In general, *L. velozianus* can be separated from the other two species, *L. demissus* and *L. weingartianus*, by a suite of morphological characters, as well as its phylogenetic relationships to the other taxa (Fig. 1). *Leptocereus velozianus* does not share any major morphological features with either *L. paniculatus* or *L. undulosus* (Figs 6, 7).

**Figure 8.**
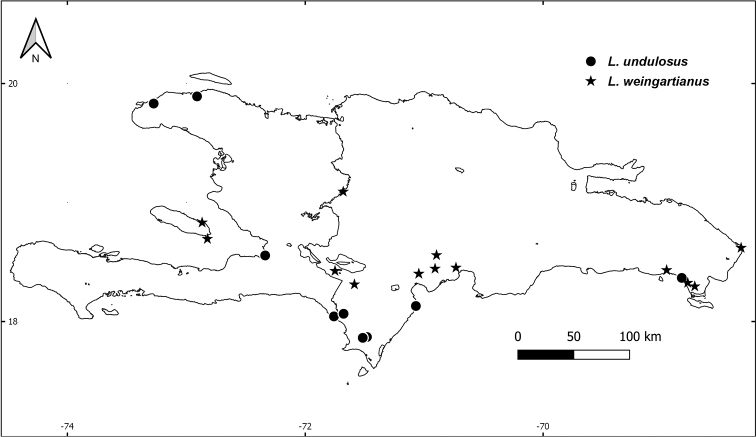
Distribution map of *L. undulosus* (black circles) and *L. weingartianus* (black stars) on Hispaniola.

As far as is known, *L. demissus* and *L. velozianus* are the most restricted species on Hispaniola, with *L. demissus* restricted to lower elevation dogtooth limestone of Parque Nacional Jaragua and the surrounding area south of the Sierra de Bahoruco, while *L. velozianus* is restricted to well-developed tropical dry forest along the north slopes of the Sierra de Bahoruco (Fig. 4). *Leptocereus paniculatus*, *L. undulosus*, and *L. weingartianus* are much more widespread, with *L. weingartianus* occurring mostly on the north island from its eastern extremity near Cabo Engaño to its western extremity in Haiti on Gonave Island in elevations ranging from near sea level to 755 m in the Sierra Martín García, Dominican Republic (see Additional specimens examined). *Leptocereus paniculatus* is quite widespread across the island and is mostly found at lower elevations from -30 m on Isla Cabritos to around 400 m near Sierra Martín García, while *L. undulosus* is mostly restricted to areas near the coast in elevations ranging from near sea level to 245 m (Fig. 8, see also additional specimens examined).

## Discussion

Our topology differs only slightly from Barrios et al. (2020) in that the CU clade was sister to the rest of *Leptocereus* here, whereas, in Barrios et al. (2020) the EPR clade was sister to the rest of *Leptocereus*. In both topologies, the D clade was nested within *Leptocereus*. Although it has been suggested that *L. undulosus* and *L. nudiflorus* may be conspecific (Anderson 2001), the multiple accessions sampled here clearly show them as genetically distinct entities, with multiple accessions of each forming clades, this likely being further driven by their reproductive isolation, being restricted to Hispaniola and Cuba, respectively. Morphological characters separating these two taxa are currently under investigation (D. Barrios unpubl. data).

Our phylogenetic results clearly demonstrate that the new species described here, *L. velozianus*, is genetically distinct from the more widespread *L. weingartianus*, and *L. demissus*, likewise, is more closely related to *L. velozianus* rather than the phenetically more similar *L. weingartianus*. Areces-Mallea (2017) mentioned that *L. demissus* is physically separated from *L. weingartianus* by the Sierra de Bahoruco. Our phylogenetic results and fieldwork support this idea, given that all *L. weingartianus* sampled were collected north of the Sierra de Bahoruco and form a well-supported clade, however, *L. weingartianus* does occur in the southern peninsula at nearly the same latitude as *L. demissus* south of Massif de la Hotte in Haiti based on the proposed neotype of *L. weingartianus* by Areces-Mallea (2017) from Cote le Fer. Interestingly, *L. weingartianus* grows alongside *L. velozianus* in the Sierra de Bahoruco; however, we have seen no evidence of hybridization between the two species. Ploidy, though, has not been examined in any species of *Leptocereus* from Hispaniola but is currently underway. *Leptocereus weingartianus* also occurs alongside *L. paniculatus* in populations near Jimaní and *L. demissus* occurs with *L. undulosus* in Parque Nacional Jaragua; however, we likewise have seen no putative hybrids among those species’ pairs. We consider that phenology and perhaps pollinator differences could be driving the lack of hybridization in *Leptocereus* on Hispaniola (although we have very little information on specific pollinators of these species), and in other parts of their ranges, *L. weingartianus* appears to occupy different ecological niches than *L. paniculatus*. For example, in the Sierra Martín García, although both species occur there, *L. paniculatus* occurs at much lower elevations than *L. weingartianus*, which begins to be found around 400 + meters (Majure et al. pers. obvs.). So, ecology also could be driving species divergence in this group of close relatives.

Below, we provide a description of the new species, *L. velozianus*, as well as a taxonomic treatment with identification key, distribution maps and photographic plates of the other four species of *Leptocereus* on Hispaniola. Updated descriptions of the other four species will be presented elsewhere (Encarnación, in prep.).

### Taxonomic treatment

#### Key to the species of *Leptocereus* on Hispaniola

**Table:** 

1	Large trees, floral buds and fruit spineless	**2**
–	Small trees, erect or sprawling shrubs, floral buds and fruit spiny (at least when immature)	**3**
2	Mature stem segments mostly spineless, usually 3–4-ribbed, fruit broadly ovate to elliptic, smooth	***L. undulosus***
–	Mature stem segments spiny, 4–5-ribbed, fruit elliptical, tuberculate	***L. paniculatus***
3	Stems 1.2–3.5 cm in diameter, ribs crenate or straight, young spines yellow to yellowish-red	**4**
–	Stems 2.7–3.7 cm in diameter, ribs strongly crenate, young spines white to cream	***L. velozianus***
4	Small trees or erect shrubs (ultimate stem segments often sprawling in age), stems mostly 4–5 ribbed, hypanthium oblong	***L. weingartianus***
–	Sprawling shrubs, stems 5–7 ribbed, hypanthium obconic	***L. demissus***

##### 1. Leptocereus velozianus

Taxon classificationPlantaeCaryophyllalesCactaceae

Clase, Y.Encarn., Peguero & Majure sp. nov.

03A984B4-6D60-5CB7-B1AF-D9736DB591F6

urn:lsid:ipni.org:names:77214766-1

Figs 2 , 3 , 4

###### Type.

República Dominicana. Prov. Independencia. Sierra de Bahoruco, Parque Nacional Sierra de Bahoruco, Puerto Escondido, Rabo de Gato, 433 m, 14 mayo 2019, *Majure 7843* (Holotype: JBSD!, Isotype: FLAS!).

###### Diagnosis.

Differing from both *L. weingartianus* and *L. demissus* by the white young spines (vs. yellowish spines) and larger stem diameter (up to 3.7 cm in diameter in *L. velozianus*). Differing from *L. demissus* by the erect, primary trunk rather than the sprawling growth form, and oblong hypanthium in *L. velozianus* rather than obconic hypanthium as in *L. demissus*.

###### Description.

Erect shrubs or small trees 2–4 m tall, ultimate stem segments often sprawling among associate vegetation in smaller individuals, or merely pendent on larger individuals, 13–40+ cm long, 2.7–3.7 cm wide, ribs 4–5 per stem, 12–17 mm deep, rib margins strongly crenate, 18–28 mm between areoles, spines white (or yellowish around the developing base), aging gray, tips brownish-red to black, 13–15 per areole, erect, central spine 16–22 mm long, longest radial spines 18–30 mm long, shortest pair of reflexed radial spines at base of areole, 3–6 mm long, a tuft of brown, crisped trichomes filling areoles; flower, including hypanthium, ca. 7.6 cm long, spiny, outer tepals green, inner tepals greenish-white, apices obtuse, anthers white, 1.3–1.5 mm long, style ca. 3 cm long, stigma included in the corolla, lobes 8, pale green, apparently at the level of the ring of anthers, nectar chamber about 8 mm wide, immature fruit, 5–7.5 × 2.6–3.4 cm (excluding dried perianth), lustrous green, spiny, areoles ca. 28–36, with 8–13 spines, those 3–12 mm long, strongly brownish-red and white banded, the tips dark brownish-red to black, seeds 3.3–3.7 × 2.3–2.5 mm, dull black (only appearing shiny when not cleaned) with colliculate-pitted surface.

###### Etymology.

The specific epithet, “*velozianus*” is given honoring the Dominican botanist Alberto Veloz, who is the Head and Curator of the Herbarium JBSD of the “Dr. Rafael M. Moscoso” National Botanical Garden of Dominican Republic. For 27 years, Veloz has dedicated his life to the study of the Hispaniolan flora and has conducted extensive fieldwork across the island, with many collections from the Sierra de Bahoruco, where this new species was found. Together with other botanists he has collected over 10,000 specimens and has published several papers on the flora in national and international journals. His publications have included different approaches, such as floristics, taxonomy, ecology and conservation. Veloz has also contributed to the formation of young botanists by involving students as part of the staff in the herbarium JBSD and through fieldwork.

###### Distribution and habitat.

*Leptocereus velozanius* grows in well-formed, seasonally dry tropical forest over limestone from around 158–433 m in elevation occurring with the following associate species: *Bursera simaruba* Sarg., *Celtis ehrenbergiana* (Klotzsch.) Liebm., *Coccoloba diversifolia* Jacq., *Consolea microcarpa* (K.Schum.) E.F.Anderson, *Cordia globosa* Kunth, *Cylindropuntia caribaea* (Britton & Rose) F.M.Knuth, *Eugenia rhombea* (O.Berg.) Krug & Urb., *Gouania lupuloides* Urb., *Guaiacum sanctum* L., *Helicteres semitriloba* Bert. ex DC., *Hybanthus havanensis* Jacq., *Krugiodendron ferreum* Urb., *Leptocereus weingartianus* (E.Hartmann) Britton & Rose, *Melochia tomentosa* L., *Opuntia repens* Bello, *Pisonia aculeata* L., *Phyllostylon rhamnoides* (J.Poiss.) Taub., *Prosopis juliflora* (Sw.) DC., *Senna angustisiliqua* (Lam.) H.S.Irwin & Barneby, *Senegalia skleroxyla* (Tussac) Seigler & Ebinger, *Tournefortia stenophylla* Urb., *Zanthoxylum nashii* P.Wilson, *Ziziphus rignonii* Delponte. Currently, the species is only known from the northwestern slope of the Sierra de Bahoruco, southwest of the town of Jimaní (Fig. 4). Given the extent of tropical dry forest in and around that area, it is very likely that more localities of this species will be found.

###### Phenology.

*Leptocereus velozianus* has been collected in flower and immature fruit during May and with mature fruit in June, July and November. Thus, it appears likely that *L. velozianus* may flower over the early to mid-summer months with fruit ripening later in the year.

###### Conservation status.

Formal evaluation of conservation status will be undertaken by Encarnación (in prep.) for *L. velozianus* based on further field work and demographic study. However, based on the currently known limited distribution of the species, the few numbers of individuals that have been observed, as well as anthropogenic activity near populations of the species, we consider that this species could be Near Threatened based on IUCN criteria. Further fieldwork will be essential for providing a comprehensive assessment of the conservation status of *L. velozianus*. Fortunately, the larger population known for this species is within the Sierra de Bahoruco National Park, so it is mostly protected from large-scale anthropogenic disturbances. Furthermore, the SDTF in that area is very well developed (i.e., non-fragmented) and should provide extra protection for the species.

###### Additional specimens examined.

Dominican Republic. Prov. Independencia. Sierra de Bahoruco, municipio Duvergé, comunidad Puerto Escondido, lugar denominado Rabo de Gato, yendo hacia Cañada de Pedro Bello, 26 jun 2013, *Clase et al. 8004* (JBSD). Sierra de Bahoruco, municipio Duvergé, Rabo de Gato, yendo hacía la cañada La Cuaba, Pedro Bello, 23 nov 2017, *Clase et al. 9931* (JBSD). Sierra de Bahoruco, municipio Duvergé, Puerto Escondido, lugar denominado Rabo de Gato, yendo hacía la Cañada de Pedro Bello, 27 jul 2017, *Clase et al. 10202* (JBSD). Sierra de Bahoruco, municipio Jimaní, yendo desde El Limón, lugar denominado Guzmán, lado Sur de la carretera, 27 jul 2017, *Clase et al. 10205* (JBSD). Sierra de Bahoruco, justo al Este de Jimaní en la carretera 46 (entre Jimaní y El Limón) en el lugar denominado Guzmán; 158 m, 14 mayo 2019, *Majure 7842* (FLAS, JBSD). Parque Nacional Sierra de Bahoruco, Puerto Escondido, Rabo de Gato; 433 m, 14 mayo 2019, *Majure 7851* (FLAS, JBSD).

##### 2. Leptocereus demissus

Taxon classificationPlantaeCaryophyllalesCactaceae

Areces Cactus Succ. J. (Los Angeles) 89: 118. 2017.

99E4D78E-62CA-5849-A042-F71900770906

Figs 4 , 5

###### Type.

Dominican Republic. Prov. Pedernales. On limestone terraces of maritime origin along both sides of the road to Pedernales, approx. 10 km southeast of Cabo Rojo, in dry forests dominated by large *Dendrocereus undulosus* trees, 22 Dec. 1998, *Areces 6812* (Holotype: JBSD-n.v.; Isotypes: NY, HAJB, HNT-n.v.).

###### Notes.

Although type material of *L. demissus* is reported to be at JBSD, HAJB, HNT, and NY (Areces-Mallea 2017), we have been unable to locate types at any of these institutions for comparative analysis for this work. Thus, we have based our knowledge on the morphological traits of this species using those characters given by Areces-Mallea (2017), geography, and the one collection from near Oviedo made by us (*Majure 5972*-see specimens examined; Fig. 5).

###### Additional specimens examined.

Dominican Republic. Prov. Pedernales. Oviedo, Sierra de Bahoruco, Municipio Oviedo, lugar denominado Fondo Paradí, Parque Nacional Jaragua, 86 m, 3 feb 2016, *Majure 5972* (DES, JBSD). Cabo Rojo, próx. al punto No. 7 de la Concesión, [no date], *Veloz et al. 945* (JBSD). A 18 km de Oviedo en la carretera hacia Pedernales, 1 mayo 1998, *Villardebó s.n.* (JBSD).

##### 3. Leptocereus paniculatus

Taxon classificationPlantaeCaryophyllalesCactaceae

(Lam.) D. R. Hunt Bradleya 9: 89. 1991.

F33BEB85-E923-5CFA-8272-3CB90F5E3D0A

Figs 4 , 6

Cactus paniculatus Lam. Encyl. [J. Lamarck & al.] 1: 540. 1785. Cereus paniculatus (Lam.) DC. Prodr. [A. P. de Candolle] 3: 466. 1828. Neoabbottia paniculata (Lam.) Britton & Rose Smithsonian Misc. Collect. 72: 3. 1921.

###### Type.

Presumably from near Cul de Sac, Haiti, where Plumier collected numerous species. Lectotype (designated by Mottram 2002), plate t.21 in Plumier, which was cited by Lamarck (1785) and thus formed part of the protologue. This includes an illustration of a fragment of a stem segment, flower and fruit.

###### Notes.

*Leptocereus paniculatus* is consistently recovered as closely related to the *L. quadricostatus/L. grantianus* clade (Fig. 1; see also Barrios et al. 2020), although the species is much more robust and treelike compared to the other two species. It is the only species on Hispaniola that has spineless, tuberculate fruits and forms large populations at lower elevations in seasonally dry tropical forest across the island (Figs 4, 6).

###### Additional specimens examined.

Dominican Republic. **Prov. Azua.** Llanura de Azua, 4 km al oeste del poblado de Tábara Abajo, en la carretera hacía Barahona, 400 m, 2 sept 1994, *García et al. 5630* (JBSD). **Provs. Azua-Barahona.** Este de Quita Coraza en el camino a Azua, 25 mayo 1982, *Zanoni & Mejía 20797* (JBSD). **Provs. Baoruco-Barahona.** A 3 km al este del poblado de Quita Coraza, 500 m, 10 dic 1994, *Perdomo & Villafaña 18* (JBSD). **Prov. Barahona**. 1.5 km al sur del poblado Fondo Negro, siguiendo un camino en dirección suroeste, 100 m, 15 oct 1994, *Camejo et al. 31* (JBSD). Barahona, 1913, *Fuertes 13.19* (NY, on 3 sheets). Sierra Martín García, approx 1.5 km al S del Higuito, 120 m, 16 dic 1995, *García et al. 6119* (JBSD). Sierra Martín García, ca 0.6 km al noreste del Cruce Vicente Noble, al costado de un arroyo seco justo al este de la Carretera 44., 108 m, 3 nov 2016, *Majure 6581* (DES, JBSD). Along road between Canoa and Puerto Alejandro, 5–20 m, 17 Oct 1982, *Proctor 39106* (JBSD). **Prov. Independencia.** Municipio Jimaní, distrito municipal Boca de Cachón, parte arriba del balneario, lugar denominado la Loma, 4 m, 6 jun 2009, *Clase et al. 5638* (JBSD). Sierra de Neiba, Ángel Félix, 400 m, 24–26 mar 1975, *Liogier et al. 22786* (JBSD). Entre Duvergé y Cabral, 3 ene 1977, *Liogier 26214* (JBSD). Independencia, Sierra de Bahoruco, al lado (sur) del Lago Enriquillo, al oeste ca. 15 km de Duvergé en la carretera 46, 5 feb 2016, *Majure 6003* (DES, FLAS, JBSD). Justo al sur de la Laguna Rincón ca. 2.5 km al oeste de La Lista y 3.4 km al este de Las Salinas a lo largo de la Carretera 46, 104 m, 13 mayo 2019, *Majure 7817* (FLAS, JBSD). Sierra de Bahoruco, ca. 1.5 km al este de El Limón en la Carretera 46 al sur del Lago Enriquillo, 43 m, 13 mayo 2020, *Majure 7840* (FLAS, JBSD). Isla Cabritos en el Lago Enriquillo, próximo al Campamento de Parques Nacionales, -30 m, 14 oct 1981, *Mejía & Pimentel 17261* (JBSD). **Prov. Montecristi.** Pepillo Salcedo (Manzanillo), al este del pueblo, en la zona donde se desarrollará el proyecto Puerto Cristal, 5 m, 5 nov 2002, *García & Gómez 7512* (JBSD). **Haiti.** [**Dept. de Nippes**]. Massif de la Hotte, western group, Anse a Veau, quaternary limestone west of town, 2 Jan 1936, *Ekman H5393* (NY). **Dept. de l’Ouest.** Vicinity of Etang Saumatre, 4–12 Apr 1920, *Leonard 3500a-b* (NY, US). Ibid, *Leonard 5344* (NY, US). Vicinity of Petionville, 350 m, 15–28 Jun 1920, *Leonard 5326* (NY).

##### 4. Leptocereus undulosus

Taxon classificationPlantaeCaryophyllalesCactaceae

(DC.) D. Barrios & Majure, Plant Syst. Evol. 306: 12. 2020.

E06CA495-FFE8-591A-BF1F-F7696A2D9133

Figs 7 , 8

Cereus undulosus DC., Prod. Syst. Nat. 3: 467. 1828. Dendrocereus undulosus (DC.) Britton & Rose, J. New York Bot. Gard. 26(310): 220. 1925. Acanthocereus undulosus (DC.) Croizat, Caldasia 2: 137. 1943.

###### Lectotype.

(designated by Barrios et al. 2020): illustration in Plumier, Plantarum americanarum fasc. 8. 187. t. 194. 1758. **Epitype.** (designated by Barrios et al. 2020): Haiti, Jean Rabel Rd., vicinity of Cabaret, Baie des Moustiques, plain west of Cabaret, 12–18 Jan 1929, *E.C. Leonard & G.M. Leonard 12085* (NY 03305544!).

###### Notes.

Although several authors have suggested that *L. undulosus* may be conspecific with *L. nudiflorus* of Cuba (Anderson 2001, Hunt et al. 2006), Barrios et al. (2020) showed that the two taxa were sister to one another and likewise were genetically divergent; however, they only sampled one accession of *L. undulosus*. We reaffirm that relationship here with one more sample of *L. undulosus* included in our phylogenetic analysis from a population near Barahona, east of the previous accession used in Barrios et al. (2020) from Parque Nacional Jaragua. Both samples form a strongly supported clade, sister to *L. nudiflorus*. More detailed morphological studies are necessary across the populations of *L. undulosus* on Hispaniola. Considering the distance between populations of the two (Cuba vs. Hispaniola), it is very likely that they are reproductively isolated. Thus, there seems to be no reason to consider these two taxa as conspecific. We anticipate that further morphological and genetic study will provide further support for the recognition of these two species.

Although not represented in our distribution map of *L. undulosus*, as there are no collections from that site, Île de la Tortue likely represents one of the larger populations of the species on Hispaniola (Peguero pers. obsv.). This reiterates the extensive fieldwork that still needs to be carried out to fully document populations of species of cacti with herbarium specimens from across the island. Cacti are often uncommonly collected because of the difficulty in preparing specimens, and thus, are generally poorly represented in herbaria (Majure et al. 2017).

###### Additional specimens examined.

Dominican Republic. **Prov. La Altagracia.** 500 m al sur del cruce hacia Bayahibe en la carretera al Parque Nacional del Este, 60 m, 22 mayo 1986, *García & Pimentel 1051* (JBSD). **Prov. Barahona.** Sierra de Bahoruco, a lo largo de Carretera 44, ca. 0.8 km al norte de Playa Azul, 13 mayo 2019, *Majure 7812* (FLAS, JBSD). **Prov. Pedernales.** Entre Pedernales y Oviedo, 150 m, 24–27 jun 1975, *Liogier & Liogier 23339* (JBSD). Oviedo, Paraje Tres Charcos, Parque Nacional Jaragua, área de sisal, 138 m, 3 feb 2016, *Majure 5974* (DES, FLAS, JBSD). Oviedo, Parque Nacional Jaragua, al suroeste de Manuel Goya y Carretera 44, Lugar denominado “Los Sisales”, cerca 14.6 km al oeste de Oviedo, 245 m, 16 nov 2016, *Majure 6586* (DES, JBSD). En la carretera hacia Las Mercedes, desde Pedernales a 2.5 km del cruce, 1 mayo 1998, *Villardebó s.n.* (JBSD). **Haiti. Dept. de l’Ouest.** Massif de la Selle, group Morne des Commissaires, Anses-a-Pitre, limestone cliff at Río Pedernales, on the road to Banane, 25 Oct 1926, *Ekman H6730* (NY). Place de la Paix, Port au Prince, no date, *Buch*? (NY). **Dept. de Nord-Ouest.** Coastal terrace between Mole St. Nicholas and Jean Rabel, 19 Jan 1995, *Areces 6790* (S). Between Port-des-Paix and Moustique, 1924, *Buch s.n.* (NY).

##### 5. Leptocereus weingartianus

Taxon classificationPlantaeCaryophyllalesCactaceae

(E.Hartmann) Britton & Rose Cactaceae 2: 77. 1920.

2BFFB8A8-AAE5-5DA7-9ADA-69E2C795BA3F

Figs 8 , 9

Cereus weingartianus E. Hartmann Monatsschr. Kakteenk. 14: 155. 1904.

###### Type.

Haiti. Lectotype (designated by Barrios and Majure, in review). Photo of type material of *L. weingartianus* in Hartmann (1904).

###### Notes.

**Figure 9.**
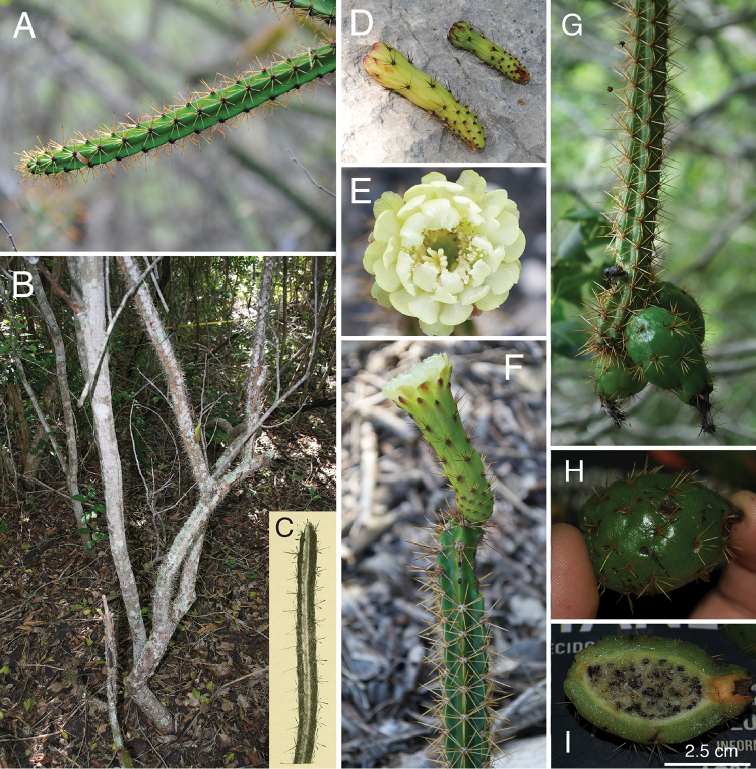
Photographic plate of *L. weingartianus***A** young developing stem showing yellowish spines **B** trunk of adult individual showing erect growth form **C** type of *L. weingartianus* from Hartmann (1904)**D** floral buds showing spiny pericarpels **E** opened flower **F** side view of open flower and associated stem showing yellowish spines **G** stem with crenate margins and spiny fruit **H** shiny, green, spiny fruit with yellowish spines, and **I** longitudinal section of fruit showing mature seeds. **A** taken from *Majure 7839***B, E, F, H, I** from *Majure 6464***D** from *Majure 7863* and **G** from *Majure 6438*. Photos by L.C. Majure.

There has been some confusion around the type of *L. weingartianus* – Areces-Mallea (2017) designated a neotype of *L. weingartianus* considering that the original type specimen is likely not extant, however, the photo of the type material in the protologue (Hartmann 1904) represents original material (Fig. 9C) and thus should serve as the type for this species. Thus, the neotype designation by Areces-Mallea (2017) from Cote le Fer, Haiti, is superfluous. Although supposedly deposited in JBSD and NY, we have been unable to find those specimens. Likewise, we have been unable to locate the other specimens of *L. weingartianus* (*Areces 5973*, *6438*, *6814*, *6815*, *6875*) cited by Areces-Mallea (2017) and supposedly at NY, so those are not cited in our specimens examined here.

###### Additional specimens examined.

Dominican Republic. **Prov. La Altagracia.** Bayahíbe, La Romana, 1–5 m, 21 Feb 1976, *Liogier & Liogier 24907* (JBSD, NY). Cabo Engaño, on coastal road, 14 May 1980, *Mejía & Zanoni 6299* (JBSD). Parque Nacional del Este, Sector Guaraguao, 21 ene 1986, *Salazar et al. 317* (JBSD). Parque Nacional del Este, en el camino hacía La Cueva de José María, Guaraguao, 1–30 m, 28 abr 2001, *Veloz & Cedeño 2648* (JBSD). **Prov. Azua.** Municipio Sabana Yegua, después del cruce del 15, yendo hacía San Juan, aprox. 600 m antes del poblado de Las Guanábanas, 241 m, 30 jul 2011, *Clase et al. 6824* (JBSD). Sierra Martín García, Sept 1976, *Liogier 25277* (JBSD). Azua, Mar 1913, *Rose et al. 3941* (NY, US). **Prov. Barahona.** Barahona, Sierra Martín García, ca. 0.6 km al noreste del Cruce de Vicente Noble, al noreste ca. 11.2 km de la Carretera 44, a lo largo del un arroyo seco, 685 m, 3 nov 2016, *Majure 6438* (DES, JBSD). Sierra Martín García, ca. 0.6 km al noreste del Cruce de Vicente Noble, al noreste ca. 11.2 km de la Carretera 44, a lo largo del un arroyo seco, 755 m, 3 nov 2016, *Majure 6464* (DES, JBSD). **Prov. Elías Piña.** Cerro San Francisco (lado del sur), afuera del poblado de Bánica al sur de Pedro Santana, 490 m, 14 mayo 2019, *Majure 7863* (FLAS, JBSD). **Prov. Independencia.** Sierra de Bahoruco, ca. 1.5 km al este de El Limón en la Carretera 46 al sur del Lago Enriquillo, 43 m, 13 mayo 2019, *Majure 7839* (FLAS, JBSD). Parque Nacional Sierra de Bahoruco, Puerto Escondido, Rabo de Gato, 433 m, 13 mayo 2019, *Majure 7850* (FLAS, JBSD). **Haiti. [Ouest].** Vicinity of Anse Galette, Gonave Island, 3–14 Mar 1920, *Leonard 3123* (NY, US). Petite Gonave Island, 9–10 Jul 1920, *Leonard 5256* (NY, US).

## Supplementary Material

XML Treatment for Leptocereus velozianus

XML Treatment for Leptocereus demissus

XML Treatment for Leptocereus paniculatus

XML Treatment for Leptocereus undulosus

XML Treatment for Leptocereus weingartianus

## Appendix 1

Species sampled in our phylogenetic analysis and GenBank accession numbers. Collector and collector number are given, or Desert Botanical Garden (DBG) accession number, as well as herbarium repository in parentheses. Herbarium acronyms follow Thiers (2017).

***Armatocereus procerus*** DBG1994 (DES-SAMN16988911); ***Calymmanthium substerile*** DBG 2015 (DES-SAMN16988912); ***Leptocereus demissus****Majure 5972* (DES, JBSD-SAMN16988913); ***Leptocereus grantianus****Majure 6972* (FLAS-SAMN16988914); ***Leptocereus maxonii****Majure 7015* (DES, FLAS, HAJB-SAMN16988915); ***Leptocereus nudiflorus****Majure 6857* (DES, FLAS-SAMN16988916), *Majure 7048* (DES, HAJB-SAMN16988917); ***Leptocereus paniculatus****Majure 6003* (DES, FLAS, JBSD-SAMN16988918), *Majure 6581* (DES, JBSD-SAMN16988919); ***Leptocereus quadricostatus****Majure 7111* (FLAS-SAMN16988920); ***Leptocereus sylvestris****Majure 6874* (DES-SAMN16988921); ***Leptocereus undulosus****Majure 6586* (DES, JBSD-SAMN16988922), *Majure 7812* (FLAS, JBSD-SAMN16988923); ***Leptocereus velozianus****Majure 7842*, *7843*, *7851* (FLAS, JBSD-SAMN16988924–SAMN16988926); ***Leptocereus weingartianus****Majure 6438* (DES, JBSD-SAMN16988930), *Majure 7839*, *7850*, *7863* (FLAS, JBSD-SAMN16988927–SAMN16988929); ***Leptocereus wrightii****Majure 7112* (DES, FLAS-SAMN16988931); ***Melocactus pedernalensis****Majure 5976* (DES, JBSD-SAMN16988932); ***Selenicereus triangularis****Majure 6480* (DES, JBSD-SAMN16988933); ***Stenocereus fimbriatus****Majure 6519* (DES, JBSD-SAMN16988934).
